# Predictive Value of Molecular Biomarkers for Oral Food Challenge Outcomes in Children with Hazelnut and Peanut Allergies

**DOI:** 10.3390/nu18030450

**Published:** 2026-01-29

**Authors:** Giulia Brindisi, Alessandro Gravina, Daniela De Canditiis, Filippo Mondì, Alessandra Gori, Francesca Olivero, Marzio Masini, Ludovica Cela, Antonio Semeraro, Anna Maria Zicari, Alberto Spalice, Maria Grazia Piccioni, Caterina Anania

**Affiliations:** 1 Department of Maternal Infantile and Urological Sciences, Sapienza University of Rome, 00161 Rome, Italy; alessandro.gravina@uniroma1.it (A.G.); filippo.mondi@uniroma1.it (F.M.); alessandra.gori85@gmail.com (A.G.); marzio.masini@uniroma1.it (M.M.); ludovica.cela@uniroma1.it (L.C.); antonio.semeraro@uniroma1.it (A.S.); annamaria.zicari@uniroma1.it (A.M.Z.); alberto.spalice@uniroma1.it (A.S.); mariagrazia.piccioni@uniroma1.it (M.G.P.); caterina.anania@uniroma1.it (C.A.); 2 Institute of Applied Calculus, National Research Council, 00185 Rome, Italy; d.decanditiis@iac.cnr.it; 3 Independent Researcher, 00100 Rome, Italy; francescaolivero31@gmail.com

**Keywords:** hazelnut, peanut, children, oral food challenge, component resolved diagnosis, precision medicine, anaphylaxis

## Abstract

**Background**: Food allergy (FA) is an emerging problem in pediatrics, with tree nuts and peanuts being frequent causes of severe reactions. Oral food challenge (OFC) remains the gold standard for diagnosing FA. However, it is a stressful treatment and not always risk-free. **Objectives**: To identify potential biomarkers, using component-resolved diagnosis (CRD) associated with OFC outcome in children with tree nut (hazelnut, walnut, almond, and pistachio) and peanut allergy, who live in central and southern Italy. **Methods**: Eighty-eight (1–18 years) children followed at the Pediatric Allergy Clinic of Policlinico Umberto I in Rome were included in this study. All patients underwent skin prick tests (SPTs), prick-by-prick (PbP) tests, and serum-specific Immunoglobulin E (sIgE) measurement to allergenic components using CRDs. **Results**: In hazelnut allergy (*n* = 60 OFCs), OFC failure occurred in 41 children. Higher sIgE levels to Cor a 8 (OR 2.04, 95% CI 1.17–3.55), Cor a 9 (OR 2.61, 95% CI 1.37–5.00), and Cor a 14 (OR 1.65, 95% CI 1.14–2.38) were all significantly associated with an increased probability of a positive OFC outcome. In peanut allergy (*n* = 30 OFCs), OFC failure occurred in 16 children. Ara h 9 was the only statistically significant predictor of OFC failure, showing a very wide confidence interval (OR, 95% CI: 1.116–484). For walnut, almond, and pistachio, sample sizes were insufficient to support inferential modeling. **Conclusions**: CRD biomarkers can stratify the likelihood of OFC reactions in pediatric FA, enhancing clinical decision-making and reducing unnecessary challenges.

## 1. Introduction

Food allergy (FA) is an abnormal immunological reaction that occurs following the ingestion of a specific food [1,2]. FA can be classified into IgE-mediated, non-IgE-mediated, and mixed forms. IgE-mediated FAs are also known as “immediate”, due to the rapid onset of symptoms (usually within a few minutes), which can include cutaneous, gastrointestinal, and respiratory manifestations, among others [3]. In 2023, the European Academy of Allergy and Clinical Immunology (EAACI) published the new guidelines for the diagnosis of FA [4]. The diagnosis of FA relies on a combination of clinical history and diagnostic tests. First-line investigations include skin prick tests (SPTs), prick-by-prick (PbP) tests, and serum-specific Immunoglobulin E (sIgE) measurements [5,6,7,8,9,10]. SPTs and PbP assess immediate hypersensitivity reactions to allergen extracts or fresh foods, whereas sIgE quantifies circulating IgE antibodies against specific allergens. Although these tests provide valuable information on sensitization, they cannot reliably predict the outcome of the oral food challenge (OFC), which remains the gold standard for FA diagnosis and for assessing tolerance acquisition after an elimination diet [11,12]. Several studies have attempted to correlate SPT or PbP size with OFC outcomes, but the results have been inconsistent and no standardized thresholds have been established [13,14].

Nut and peanut allergies are a major concern in pediatric populations due to their prevalence and the risk of severe reactions. In Western countries, the prevalence of nut allergy ranges from 1% to 2%, with hazelnut and peanut among the most common allergens [14,15,16,17,18,19,20]. In Italy, nut allergy is the second leading cause of food anaphylaxis, with hazelnut allergy affecting approximately 0.2% of children [21,22]. Peanut allergy is also increasingly prevalent worldwide, with estimates ranging from 0.1% to 2.2% depending on the population and diagnostic method [23,24,25]. Clinical manifestations of these allergies vary, but cutaneous reactions are the most common, followed by gastrointestinal and, more rarely, respiratory symptoms; anaphylaxis may occur in a substantial proportion of cases [26,27,28]. These allergies are often persistent and difficult to outgrow, highlighting the need for effective risk stratification.

In the precision medicine era, molecular diagnostics may represent a valuable tool for pediatric allergists to guide decisions about whether to perform an OFC; however, pediatric studies on this topic remain scarce [11]. Component-resolved diagnostics (CRD) enables the identification of specific molecular allergens and may improve risk assessment in FA management. In this study, we analyzed CRD profiles for hazelnut (Cor a 1, Cor a 8, Cor a 9, and Cor a 14), peanut (Ara h 1, Ara h 2, Ara h 3, Ara h 8, and Ara h 9), and, in some cases, walnuts, almonds, and pistachios in children who live in central and southern Italy [29,30,31,32,33,34,35,36,37,38].

Hazelnut molecular components differ in clinical relevance: Cor a 1 (Cor a 1.0401) is a labile protein (PR-10) associated with birch pollen cross-reactivity and oral allergy syndrome, whereas Cor a 8 (LTP) and the storage proteins Cor a 9 (11S globulin) and Cor a 14 (2S albumin) are heat- and digestion-stable and are associated with systemic and severe allergic reactions. Sensitization patterns vary geographically: in Northern Europe, Cor a 1 predominates due to birch exposure, while in Mediterranean populations, Cor a 8, Cor a 9, and Cor a 14 are more prevalent, especially in children [39]. Peanuts contain several allergenic components, among which Ara h 1, Ara h 2, and Ara h 3, and related storage proteins are traditionally considered the major allergens because of their resistance to heat and digestion. In contrast, Ara h 8 belongs to the Bet v 1 homologues, while Ara h 9 is a lipid transfer protein (LTP), reflecting different allergenic pathways [40].

Even in a few cases, we also tested CRD for walnuts, almonds, and pistachios. The CRD for walnuts examined in our study were Jug r 1 (2S albumin) and Jug r 3 (nsLTP) [33,34,35]. IgEs specific to Jug r 1 were detected in most walnut-allergic patients in a US-based study, while sensitization to Jug r 3 seems to be prevalent among Italian patients [36].

Regarding almond allergy, eight native almond allergens have been characterized according to their biochemical function, and Pru du 3 is the nsLTP [37]. Of the four allergens identified for pistachio, the major allergen is Pis v 1 (2S albumin) [38].

This study aimed to identify biomarkers associated with a higher risk of OFC failure in children with tree nut and peanut allergies followed at our center in Rome and living in central and southern Italy. We hypothesized that specific molecular sensitization profiles, as assessed by CRD, could predict OFC outcomes and guide clinical decision-making, thereby reducing the need for unnecessary and stressful challenges.

## 2. Materials and Methods

### 2.1. Study Design

A prospective observational study was conducted in 2024 at the Pediatric Allergy and Immunology Clinic of Policlinico Umberto I in Rome. The study aimed to identify molecular biomarkers predictive of OFC outcomes. Assessments included clinical history, SPTs, and PbP testing CRD and OFC. OFC was conducted in accordance with the recommendations of the EAACI [41] and the PRACTALL consensus on food challenge tests [42]. The study design and procedures have been approved by the Ethics Committee of Policlinico Umberto I in Rome. Informed consent was obtained from parents/legal guardians.

### 2.2. Participants

Eighty-eight children (1–18 years) with IgE-mediated FA to hazelnut, peanut, walnut, almond, or pistachio were included in the study. Inclusion criteria were: age 0–18 years, diagnosis confirmed by SPT, PbP, and CRD, and willingness to undergo OFC. Exclusion criteria were: active infection, chronic disease, or recent use of antibiotics, antihistamines, or corticosteroids (within the previous 15 days).

### 2.3. Component Resolved Diagnosis (CRD)

All the patients underwent a blood sample to detect sIgE related to the allergenic molecules listed below:

- Hazelnut: Specific CRDs are Cor a 1 (PR-10), Cor a 8 (nsLTP), Cor a 9 (11S globulin), and Cor a 14 (2S albumin) [43]; Bet v 1 (PR-10) and Bet v 2 (PR-10) [44].
- Peanut: Specific CRDs are Ara h 1 (7S protein), Ara h 2 (2S albumin), Ara h 3 (11S globulin), Ara h 8 (PR-10), and Ara h 9 (nsLTP) [31,32].
- Walnut: Specific CRDs are Jug r 1 (2S albumin) and Jug r 3 (nsLTP) [33,34,35].

All measurements were performed using a fluorescence enzyme immunoassay (FEIA) on a Phadia™ 250 laboratory system platform with capsulated cellulose polymer solid-phase ImmunoCAP^®^ allergens (Thermo Fisher Scientific, Phadia AB, Uppsala, Sweden), with the exception of almond and pistachio, for which sIgE tests were requested.

Specific IgE values were analyzed on their original scale. Results were expressed in kUA/L, and a cutoff of 0.1 kUA/L was used to define positivity.

### 2.4. Total IgE

Total IgE was measured using a standardized fluoroenzyme immunoassay (FEIA) on the ImmunoCAP system (Thermo Fisher Scientific). The assay relies on a solid-phase immunoassay in which serum IgE binds to immobilized anti-IgE antibodies, and fluorescence intensity, measured after enzyme labeling, is proportional to IgE concentration.

### 2.5. Oral Hazelnut Challenges

All patients underwent one or more OFC on non-consecutive days. Expert pediatric allergologists and trained nurses conducted all the OFCs [45]. The test was considered negative if the patient consumed all the prescribed doses of the offending food without experiencing any reaction. It was considered positive if, during the test, objective clinical signs and subjective, moderate-to-severe symptoms of increasing severity appeared, preventing the patient from consuming all prescribed doses of the culprit food.

For hazelnut, the cumulative dose of protein progressively and incrementally given every 15–20 min was as follows: 0.15 mg, 0.75 mg, 1.5 mg, 7.5 mg, 15 mg, 75 mg, 150 mg, and 300 mg [46].

For peanut, the cumulative dose of protein given progressively was 1.3 mg, 2.6 mg, 6.5 mg, 13 mg, 39 mg, 78 mg, 156 mg, and 312 mg [47].

For walnuts, the cumulative dose of protein progressively and incrementally given was 0.75 mg, 1.5 mg, 3.75 mg, 7.5 mg, 15 mg, 22.5 mg, 45 mg, 90 mg, 180 mg, and 300 mg [48].

For almonds, the cumulative dose of protein given progressively was 1.05 mg, 2.1 mg, 5.25 mg, 10.5 mg, 21 mg, 31.5 mg, 63 mg, 126 mg, 252 mg, 420 mg, and 840 mg [48].

For pistachio, the cumulative dose of protein progressively and incrementally given was 1 mg, 2 mg, 5 mg, 10 mg, 20 mg, 30 mg, 60 mg, 120 mg, 240 mg, 400 mg, and 800 mg [48].

The OFC was considered positive and therefore classified as a failure if clinical symptoms appeared during the administration of incremental doses of the test food, according to the EAACI recommendation [41] and the PRACTALL consensus on food challenge tests [42].

### 2.6. Statistical Analysis

Data were collected using Microsoft Excel and analyzed using the R software package version 4.4.3. The analysis was conducted in two phases. In the first phase, descriptive statistics were used to summarize and visualize the experimental results. Two summary tables were generated: one describing the sample’s demographic characteristics, and the other reporting the empirical frequencies of observed symptoms.

Since each child underwent at least one OFC, the total number of tests exceeded the number of children. For each allergen tested, the subgroup of children exposed to that allergen was analyzed, and a boxplot was used to compare wheal diameters between those who passed and those who failed the OFC. A boxplot displays a dataset’s distribution through five summary statistics (minimum, Q1, median, Q3, and maximum). The box represents the interquartile range (IQR), with the median marked inside; whiskers extend to 1.5× IQR, and outliers are shown as separate points. When one group has a single observation, the boxplot reduces to a horizontal line at the median.

In the second phase, the analysis aimed to evaluate whether molecular IgE profiles could predict OFC outcomes. The analysis focused on allergens with sufficient data, namely hazelnut and peanut. For each allergen, the OFC outcome was modeled using logistic regression, with the binary response variable indicating challenge failure (1) or tolerance (0) and allergen-specific IgE components as predictors. The objective was to identify IgE components associated with the probability of a positive OFC and to quantify their effects.

## 3. Results

In the first phase of descriptive analysis, the demographic characteristics of the sample are summarized in Table 1. Table 1 also reports the number of tests performed per child and the total number of OFCs conducted for each of the five allergens considered: hazelnut, peanut, walnut, almond, and pistachio. Overall, 117 OFCs were performed; 17 children underwent two challenges and 6 underwent three (Table 1). Allergen-specific logistic regression models were fitted separately, as the number and type of available IgE components differed across foods. The IgE components considered were Cor a 1, Cor a 8, Cor a 9, Cor a 14, Bet v 1, and Bet v 2 for hazelnut; Ara h 1, Ara h 2, Ara h 3, Ara h 8, and Ara h 9 for peanut; Jug r 1 and Jug r 3 for walnut; Pru du 3 for almond; and Pis v 1 for pistachio.

Due to limited OFC sample sizes—60 for hazelnut, 30 for peanut, 15 for walnut, 8 for almond, and 4 for pistachio reliable regression models could be fitted only for hazelnut and peanut. Given the relatively small number of observations compared with the number of predictors (6 for hazelnut and 5 for peanut), Firth’s bias-reduced logistic regression was used instead of standard logistic regression to obtain more reliable coefficient estimates, confidence intervals, and *p*-values, and to reduce small-sample bias. Descriptive statistics for binary variables indicating the presence or absence of specific symptoms are presented in Table 2.

**Figure 1:**
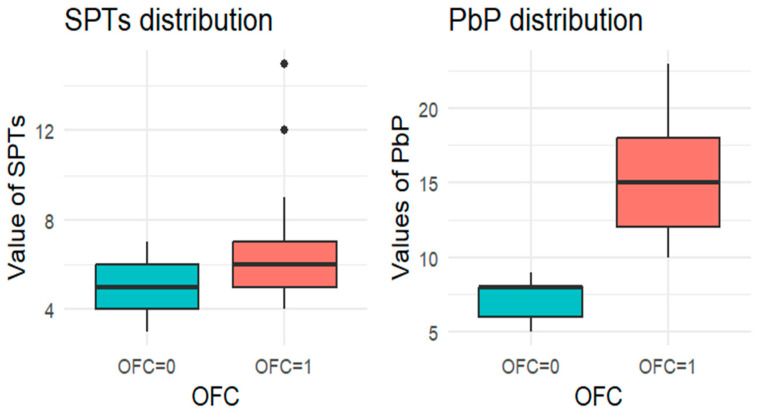
BOXPLOT of the skin prick test (SPT) and prick-by-prick (PbP) test distribution in hazelnut. SPT: skin prick tests; PbP: prick by prick; OFC = 1 failure of tolerance; OFC= 0 acquisition of tolerance.

**Figure 2:**
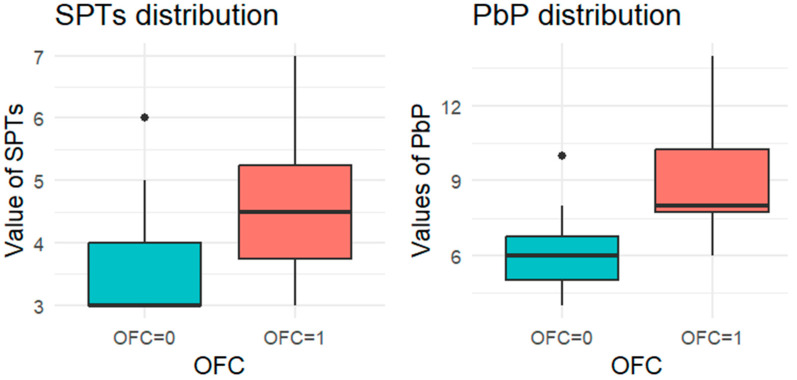
BOXPLOT of the skin prick test (SPT) and prick-by-prick (PbP) test distribution in peanut. SPT: skin prick tests; PbP: prick by prick; OFC = 1 failure of tolerance; OFC= 0 acquisition of tolerance.

**Figure 3:**
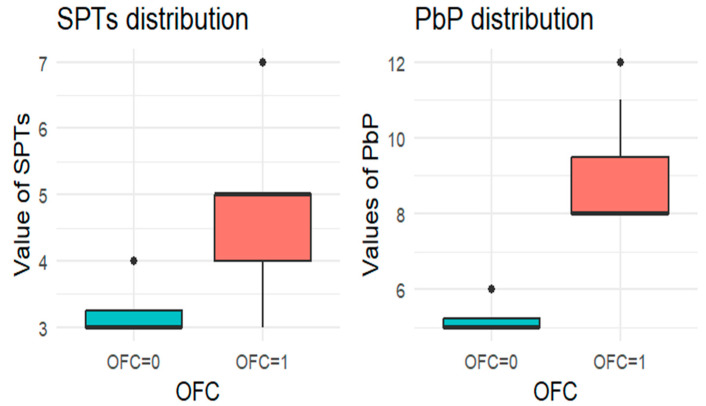
BOXPLOT of the skin prick test (SPT) and prick-by-prick (PbP) test distribution in walnut. SPT: skin prick tests; PbP: prick by prick; OFC = 1 failure of tolerance; OFC= 0 acquisition of tolerance.

**Figure 4:**
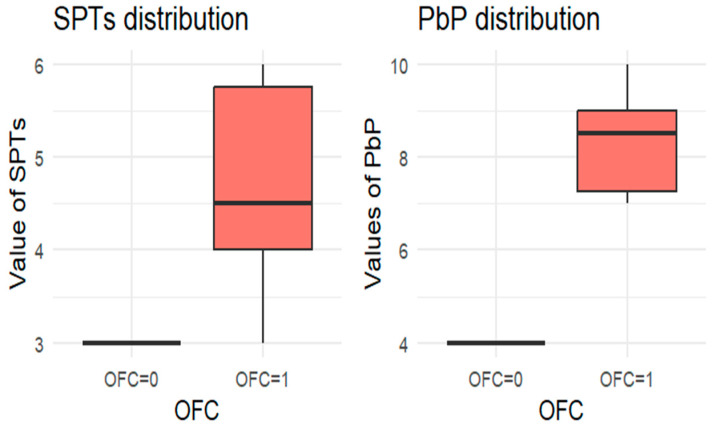
BOXPLOT of the skin prick test (SPT) and prick-by-prick (PbP) test distribution in almond. SPT: skin prick tests; PbP: prick by prick; OFC = 1 failure of tolerance; OFC= 0 acquisition of tolerance.

**Figure 5:**
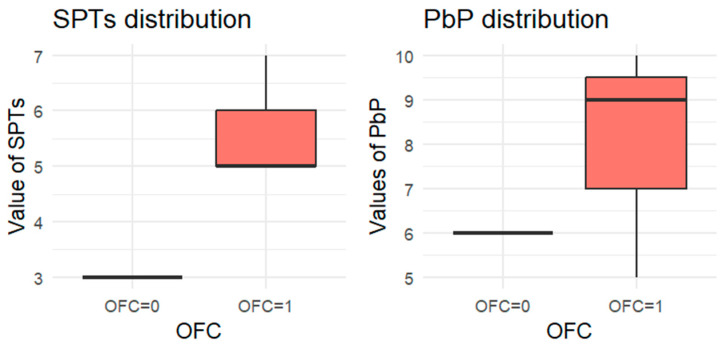
BOXPLOT of the skin prick test (SPT) and prick-by-prick (PbP) test distribution in pistachio. SPT: skin prick tests; PbP: prick by prick; OFC = 1 failure of tolerance; OFC= 0 acquisition of tolerance.

For each allergen, the subgroup of children who underwent the corresponding OFC was identified and divided into two groups: those who passed the test (OFC = 0) and those who failed (OFC = 1). Figure 1, Figure 2, Figure 3, Figure 4 and Figure 5 present boxplots of the wheal diameters for each allergen, comparing the two groups across both the SPTs and the PbP tests.

The two groups were not compared using an unpaired Student’s *t*-test for three main reasons: (1) the differences between groups are visually evident from the boxplots (Figure 1, Figure 2, Figure 3, Figure 4 and Figure 5); (2) the relationship between wheal size (from SPT and PbP) and OFC outcome is already well established and not the focus of this study; and (3) for almond, walnut, and pistachio, the sample size was too small to justify a hypothesis test.

In the following, we report the results of the second, inferential phase of the analysis, which are summarized in Table 3 and Table 4.

**Figure 6:**
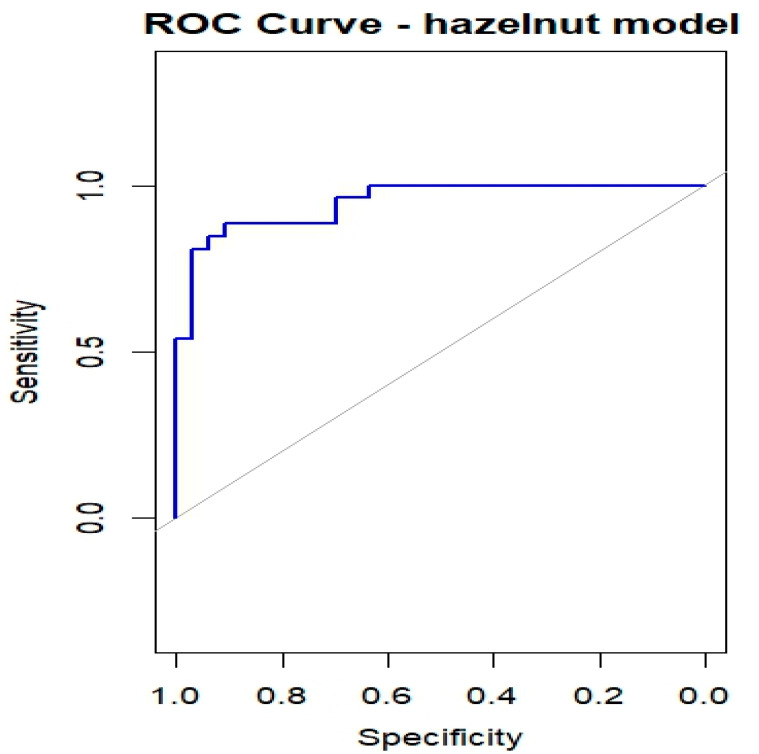
ROC curves for OFC outcome in children with Hazelnut allergy.

Table 3 summarizes the results of the bias-reduced logistic regression for hazelnut, reporting the estimated coefficients, standard errors, odds ratios (ORs), 95% confidence intervals, and *p*-values for the six predictors (Cor a 1, Cor a 8, Cor a 9, Cor a 14, Bet v 1, and Bet v 2). Of the 60 subjects who underwent the hazelnut OFC, one had missing values and was excluded from the regression analysis; therefore, 59 subjects were included, of whom 26 (44.1%) experienced OFC failure (OFC = 1) and 33 (55.9%) successfully completed the OFC (OFC = 0). Cor a 9 and Cor a 14 emerged as the most relevant predictors (both *p* < 0.01), with positive regression coefficients indicating that higher sIgE levels are associated with an increased probability of OFC failure. Cor a 8 was also significantly associated with the outcome (*p* < 0.05), whereas Bet v 2 showed a borderline association and may contribute to risk stratification despite not reaching conventional statistical significance. The ROC curve shown in Figure 6 yielded an AUC of 0.949, indicating a high discriminative ability of the model in this dataset. However, given the limited sample size relative to the number of predictors, these results should be interpreted with caution, as model performance may be overestimated and requires validation in independent cohorts.

Table 4 summarizes the results of the bias-reduced logistic regression for peanut, reporting the estimated coefficients, standard errors, odds ratios (ORs), 95% confidence intervals, and *p*-values for the five predictors (Ara h 1, Ara h 2, Ara h 3, Ara h 8, and Ara h 9). Among the 30 patients who underwent a peanut OFC, 16 (53%) experienced OFC failure (OFC = 1) and 14 (47%) successfully tolerated the challenge (OFC = 0). Ara h 9 emerged as the only statistically significant predictor, with a positive coefficient indicating that higher sIgE levels are associated with an increased likelihood of OFC failure. In this small dataset, the near-complete separation induced by Ara h 9 resulted in an apparent perfect in-sample discrimination (AUC = 1), with the ROC curve lying entirely at the upper boundary. This reflects the strong discriminatory ability of Ara h 9 in this cohort but also highlights the impact of the limited sample size and potential overfitting; therefore, these results should be interpreted with caution and validated in independent populations.

## 4. Discussion

Precision medicine is increasingly important in diagnosing and managing FA. CRD complement clinical assessment by identifying co- or cross-sensitizations, improving diagnostic accuracy, and informing patient management, particularly for predicting severe reactions during OFCs [49].

Although OFC remains the gold standard for FA diagnosis [11], it is time-consuming, costly, and carries a risk of severe reactions. Identifying CRD biomarkers predictive of OFC outcomes is therefore crucial. In this study, we identified specific biomarkers for hazelnut and peanut allergies that were associated with increased OFC failure. In contrast, small sample sizes for walnuts, almonds, and pistachios limited the identification of reliable biomarkers for these nuts.

### 4.1. Hazelnut

Our study confirms, in agreement with the existing literature, that Cor a 9 and Cor a 14 are the strongest predictors of a positive OFC outcome in children with hazelnut allergy, with Cor a 8 also emerging as a significant predictor. Children sensitized to these components are unlikely to pass the OFC. Among cross-reactive molecules, Bet v 2 showed a trend toward statistical significance and may support clinical decision-making when considered in combination with other relevant hazelnut proteins [40].

To date, only a limited number of pediatric studies have investigated the molecular profiles of children who fail OFCs to hazelnut. Overall, the available evidence consistently highlights the central role of storage proteins, particularly Cor a 14 and Cor a 9, in predicting clinically relevant hazelnut allergy in children.

The multicenter European study conducted by Datema et al. has shown that sensitization to Cor a 9 and Cor a 14 is positively associated with severe allergic reactions during double-blind, placebo-controlled food challenges, whereas Cor a 1 is generally linked to milder or absent reactions. Diagnostic models combining CRD with clinical symptoms outperform CRD alone, particularly in identifying children at low risk of severe reactions [50].

Other pediatric cohorts have confirmed Cor a 14 as the most reliable diagnostic marker, with defined IgE cutoff values accurately identifying the majority of hazelnut-allergic children and helping to predict OFC outcomes. Cor a 9 also contributes significantly, particularly in children who fail OFCs, whereas Cor a 1 and Cor a 8 show little discriminatory value. The relevance of Cor a 14 remains consistent even in the presence of a coexisting peanut allergy or sensitization to cross-reactive pollens, without interfering with challenge results [51].

Smaller studies have additionally suggested a potential role for Cor a 11 in determining reaction severity, although its relevance appears to be cohort-specific [46].

Moreover, geographical differences have emerged, with studies in Japanese children emphasizing the predictive value of high Cor a 9 and low Cor a 1 levels [52], whereas research from the Eastern Mediterranean region reinforces Cor a 14 as the key biomarker distinguishing reactive from tolerant children [53].

Cutoff values for Cor a 9 and Cor a 14 have demonstrated high specificity for severe hazelnut allergy, particularly in pediatric populations [54].

Systematic review encompassing multiple pediatric studies have confirmed that low levels of Cor a 14 and/or Cor a 9 are associated with tolerance, whereas high levels indicate a persistent and potentially severe allergy. Overall, Cor a 14 stands out as the most sensitive and specific biomarker for pediatric hazelnut allergy, clearly outperforming other hazelnut components and cross-reactive allergens in diagnostic accuracy [55].

### 4.2. Peanut

In our study, sensitization to Ara h 9 was directly associated with increasing blood sIgE levels and a higher probability of failing a peanut OFC.

Several large pediatric studies in the literature have emphasized the predominant role of Ara h 2 and Ara h 6 in predicting peanut OFC failure and reaction severity. These studies consistently identify Ara h 2, often in combination with Ara h 6, as the most accurate biomarker for peanut allergy and for reducing the need for OFCs. In these cohorts, Ara h 9 has generally shown limited predictive value for OFC outcomes, despite being present in a substantial proportion of allergic children [56]. Additional investigations have demonstrated that Ara h 2 levels correlate with OFC positivity but not necessarily with reaction severity, reinforcing the continued usefulness of OFCs in assessing tolerance [57]. Advanced diagnostic approaches, including basophil activation tests (BAT) combined with CRD, have further improved predictive accuracy, particularly when evaluating reactivity to Ara h 2 and Ara h 6 [58]. However, sensitization patterns vary significantly across populations and geographic regions. Several Italian studies have shown that Ara h 9 is the dominant peanut allergen in Italy, particularly in central and southern regions, and that its prevalence increases with age. In contrast, storage proteins such as Ara h 2 are more commonly detected in younger children and in northern Italy. Because our study population was recruited in Rome, largely representing central and southern Italy, the prominent role of Ara h 9 observed in our cohort is consistent with these regional sensitization patterns [25]. Therefore, in our pediatric cohort of children living in central and southern Italy, the prevalence of Ara h 9 is higher than that of Ara h 2.

The Italian study conducted by Calamelli et al. also supports these findings. In a sample of 48 children and adolescents with peanut-specific IgE, 58% were sensitized to Ara h 9, while 27% were sensitized to Ara h 2. Among younger children (2–5 years), seed storage proteins (such as Ara h 2) were more prevalent; however, with increasing age, Ara h 9 became dominant [59]. In conclusion, while Ara h 2 remains a key peanut allergen and a robust predictor of OFC failure in many international pediatric cohorts, it is not necessarily the most prevalent sensitizing component in the Italian population. In this context, Ara h 9 appears to play a pivotal role, particularly among adolescents and adults and in central and southern Italy, underscoring the importance of considering geographic and population-specific differences when interpreting component-resolved diagnostics.

### 4.3. Hazelnut and Peanut

Finally, few studies, including ours, have focused on children allergic to both peanuts and hazelnuts and who underwent OFC.

Among these studies, one was a prospective multicenter investigation conducted by Bayer et al. The authors performed oral challenges with hazelnut 143 children, as well as 210 with peanut. The values of Cor a 14 and Ara h 2, with an area under the curve of 0.89 and 0.92, respectively, discriminated allergic children from tolerant ones, more accurately than sIgE. Therefore, Cor a 14 and Ara h 2 appear to be useful biomarkers in clinical practice for estimating the probability of a positive OFC and for reducing unnecessary OFC [60].

Another multicenter study conducted by Grabenhenrich L. et al. among children with peanut or hazelnut allergy undergoing OFC evaluated whether the ratio of component-specific IgE to total IgE improved the prediction of challenge outcome. Specific IgE levels for peanuts, hazelnuts, and their components (Ara h 1, Ara h 2, Ara h 3, Ara h 8, Cor a 1, Cor a 8, Cor a 9, and Cor a 14) as well as total IgE were measured using ImmunoCAP. Specific IgE-to-total IgE ratios were compared with individual sIgE levels for discrimination and prediction of OFC.

The authors concluded that the ratios (component-specific IgE/total IgE) were no better than single-component specific IgE measures, and that the most useful sIgEs remained Ara h 2 and Cor a 14 [61].

### 4.4. Strengths and Weaknesses

To the best of our knowledge, this study is the first in Italy to analyze pediatric populations allergic to both hazelnuts and peanuts for molecular biomarkers predictive of OFC outcomes. While CRD shows promising predictive performance, it cannot fully replace OFCs, which remain essential. Limitations of this study include geographic variability, cross-reactivity (e.g., Bet v 1 with Cor a 1), and a lack of universally applicable cut-off values. These factors highlight the need for individualized interpretation of CRD in the context of clinical history and skin test results. Lastly, another limitation of the study is the small sample size. Therefore, larger studies are needed for external validation in independent cohorts and to confirm and extend our findings, particularly for pistachios, almonds, and walnuts.

## 5. Conclusions

Notably, in our cohort of Italian patients allergic to both hazelnuts and peanuts, we observed a predominant pattern of sensitization to storage proteins. Although this is the only study of this kind in Italy, it remains among the very few conducted in Europe on a pediatric population allergic to both hazelnuts and peanuts to identify a biomarker that could predict the outcome of an OFC. For hazelnut, we found a statistically significant association between high values of storage proteins Cor a 9, Cor a 14, and Cor a 8 and OFC failure. For peanuts, high Ara h 9 values were associated with a higher likelihood of failure to achieve tolerance. No conclusions could be drawn for walnut, pistachio, and almond, given the small number of patients involved.

Although several studies support the use of molecular components (such as Ara h 9, Ara h 2 and Cor a 14, Cor a 9, or Cor a 8) as useful predictors of OFC outcomes in children, it is essential to underline that these markers can help stratify risk, guide decisions about whether or not to perform an OFC, and potentially reduce the number of challenges required, but they do not entirely replace the need of OFC in all cases, particularly when confirmation of allergy or assessment of its severity is needed.

The future of pediatric allergy research should focus on longitudinal biomarker studies that integrate CRD into guidelines alongside OFC, which remains the gold standard. In the near future, testing multicomponent diagnostic algorithms in larger pediatric populations will be essential to improve risk stratification and clinical decision-making.

## Figures and Tables

**Table 1:** Demographic details of the study population in terms of frequency and percentage.

Variables		Frequency	Percentage
Age	1–6 (years)	15	17%
	6–12 (years)	45	51%
	12–18 (years)	26	32%
Gender	Female	39	44%
	Male	49	56%
Family history of allergy	Yes	47	53.5%
	No	41	46.5%
Number of prior AR	0	4	4.5%
	1	55	62.5%
	2	18	20.5%
	3	7	8%
	4	3	3.5%
	5	1	1%
Total IgE	115–696 (kU/L)	61	69%
	696–1256 (kU/L)	24	27%
	1256–1826 (kU/L)	3	4%
Allergen tested on OFC	Hazelnut	60	51%
	Peanut	30	26%
	Almond	8	7%
	Walnut	15	13%
	Pistachio	4	3%
	Total	117	
Number of OFC per children	1	65	74%
	2	17	19%
	3	6	7%
Age of first reaction	0–42 (months)	33	44.6%
	42–84 (months)	23	31%
	84–126 (months)	10	13.4%
	126–170 (months)	8	11%

AR: allergic reaction; OFC: oral food challenge; IgE: immunoglobulin E.

**Table 2:** Frequencies of symptoms among the enrolled patients.

Variable	Overall = 88
Oral allergy syndrome	32 (36%)
Contact skin reaction	10 (11%)
Urticaria/angioedema	35 (40%)
Other skin rash	5 (5%)
AD Flare	2 (2%)
Rhinitis/sneezing	8 (10%)
Dysphonia/pruritus/throat constriction	22 (25%)
Cough	70 (20%)
Bronchospasm	24 (27%)
Dyspnea	9 (11%)
Desaturation	0
Abdominal pain	26 (30%)
Vomiting	24 (27%)
Diarrhea	11 (12%)
Hypotension	4 (4%)
Syncope	0
Anaphylaxis	32 (36%)
History of AR/conjunctivitis	52 (60%)
History of asthma	35 (40%)
History of AD	60
Tolerates hazelnut spread	40
Tolerates allergen traces	50

AD: atopic dermatitis; AR: allergic rhinitis.

**Table 3:** Logostic regression for hazelnut.

Variable	Coeff	SE	OR	CI_Lower	CI_Upper	*p*_Value
(Intercept)	−4.360	1.224	0.013	0.001	0.141	0.00036645
Cora1	0.023	0.251	1.023	0.625	1.674	0.92772000
Cora8	0.712	0.283	2.038	1.171	3.547	0.01179600
Cora9	0.961	0.331	2.613	1.365	5.003	0.00374980
Cora14	0.498	0.187	1.645	1.139	2.375	0.00789710
Betv1	−0.022	0.109	0.978	0.790	1.211	0.83788000
Betv2	0.789	0.434	2.201	0.940	5.153	0.06919700

**Table 4:** Logistic regression for peanut.

Variable	Coeff	SE	OR	CI_Lower	CI_Upper	*p*_Value
(Intercept)	−4.830	2.451	0.008	0.000	0.974	0.048744
arah1	−2.975	1.774	0.051	0.002	1.652	0.093555
arah2	−3.656	2.451	0.026	0.000	3.152	0.135790
arah3	2.460	1.821	11.709	0.330	415.847	0.176750
arah8	0.866	0.656	2.378	0.657	8.606	0.186780
arah9	3.146	1.549	23.246	1.116	484.189	0.042268

## Data Availability

The raw data supporting the conclusions of this article will be made available by the authors on request.
